# A Comparative Evaluation of ABO Blood Group Antibody Titer Using Normal Saline, Low Ionic Strength Solution, and AB Plasma

**DOI:** 10.7759/cureus.90458

**Published:** 2025-08-19

**Authors:** Vikrant Sharma, Aakriti Puri, Babita Raghuwanshi, Ananthakrishnan M

**Affiliations:** 1 Transfusion Medicine and Blood Bank, All India Institute of Medical Sciences, Bhopal, Bhopal, IND; 2 Community and Family Medicine, All India Institute of Medical Sciences, Bhopal, Bhopal, IND

**Keywords:** abo antibody titer, ab plasma, anti-a antibody, anti-b antibody, low ionic strength solution (liss), normal saline 0.9%

## Abstract

Background

Acute hemolytic transfusion reactions, hemolytic disorders in fetuses and neonates, and hemolysis in incompatible allogeneic hematopoietic stem cell transplantation are predominantly induced by antibodies in the ABO blood group system. Environmental factors appear to exert the most significant influence on the concentrations of isoagglutinin A and B antibody molecules, which may be classified as IgG, IgM, or IgA. The current study aimed to evaluate the ABO isoagglutinin A and B titers of whole blood donors.

Methods

This observational cross-sectional study was conducted at the All India Institute of Medical Sciences, Bhopal, India. We evaluated ABO isoagglutinin A and B titers in 199 healthy blood donors (Groups A, B, and O). Donor selection was based on national guidelines. Antibody titrations were performed using 0.9% normal saline (NS), low ionic strength solution (LISS), and AB plasma, followed by a comparative analysis.

Results

Antibody titration was done with all blood group donors with three different diluent media. Spearman's rank correlation coefficient (r) is used to assess the strength and significance of the relationship between titer measurements. A p-value under 0.05 was considered statistically significant for all analyses. A strong positive correlation was observed between NS and LISS (r = 0.828, *p* < .001), indicating high concordance between these two methods. Moderate correlations were found between AB plasma and both NS (r = 0.320) and LISS (r = 0.417), suggesting that AB plasma may yield slightly variable results compared to the other two techniques. All correlations were statistically significant (*p* < .05) in the anti-A titer of Blood Group B.

Conclusion

Among the three diluent media-normal saline, low ionic strength solution, and AB plasma-the low ionic strength solution yielded the most favorable titration results, followed by normal saline. Either diluent may be utilized for ABO antibody titration, contingent upon sensitivity. AB plasma produces inferior outcomes in comparison to other types. However, further multicenter studies are required to validate our results.

## Introduction

Karl Landsteiner found the ABO blood group in 1900 [1]. Since then, about 600 other types of antibodies and red cell antigens have been identified, allowing transfusion medicine to progress [2].

Acute hemolytic transfusion reaction, hemolytic illness in fetuses and neonates, and hemolysis in incompatible allogeneic hematopoietic stem cell transplantation are all primarily caused by antibodies within the ABO system. Moreover, ABO incompatible liver, heart, and kidney transplants may lead to hyperacute graft rejection [3].

Transfusing O blood group to patients of any group has persisted since World War II, despite the potential for severe red cell damage in group A recipients receiving group O plasma transfusions. Acute hemolysis may occur more frequently than is thought and has been documented after transfusion of another group single donor platelet (PLT) concentrates. Furthermore, a positive direct anti-globin test (DAT), hemoglobinemia, jaundice, growing anemia, spontaneous agglutination, and a rise in the patient's red blood cells' osmotic fragility are all consequences of incompatible plasma [4].

The amounts of isoagglutinin A and B antibody molecules, which can be IgG, IgM, or IgA, appear to be primarily influenced by environmental variables. All three are present in some sera, while IgM predominates in non-stimulated individuals. Incompatible transfusions or additional vaccinations during pregnancy can alter the properties of isoagglutinin A or B. They can be identified serologically by increases in titers, agglutinin avidity, and hemolytic activity; they are most active at 37ºC. Group O individuals possess two distinct isoagglutinins, A and B, as well as a cross-reacting antibody, anti-A, B, predominantly of the IgG class [5].

Plasma incompatibility affects 10-40% of platelet transfusions in the United States. Simultaneously, the University of Michigan Hospitals also did prospective testing of Group O apheresis donors with the use of at least 64 as a critical titer for high-titer isoagglutinin presence [6].

Likewise, there is a paucity in blood and organ donors who are identical. Blood bank techniques are required in both cases to reduce the impact of ABO antibodies. However, two barriers to the appropriate selection of donors are the non-availability of an appropriate technique and a critical titer that can reliably identify safe donors for both clinical situations. A critical titer refers to a specific antibody level in the donor's blood that indicates a significant risk of complications, particularly acute hemolytic reaction, hemolytic disease of the fetus and newborn (HDFN) [7].

The current study aimed to assess the ABO isoagglutinin A and B titers among donors in order to avoid hemolytic transfusion reactions and compile a registry of donors with low and high ABO antibody titers.

## Materials and methods

A cross-sectional observational study was undertaken from March 2025 to June 2025 at the Immunohematology area of the Department of Transfusion Medicine and Blood Centre at the All India Institute of Medical Sciences, Bhopal, Madhya Pradesh, India. Every donor who came to the blood center had their eligibility to donate blood checked in accordance with the 1955 Drug and Cosmetic Act. Daily samples were collected and processed on the same day. Prior to beginning the investigation, Institutional Ethics Committee (IEC) approval was acquired (IHEC-LOP/2025/|P049), dated March 20, 2025. All donor information at our blood center is maintained in confidentiality.

Inclusion and exclusion criteria

Following the acquisition of written informed consent, donors who satisfied the eligibility conditions [8] were incorporated into the study in compliance with the 1940 Drugs and Cosmetics Act and the 1945 Regulations. Every donor who was accepted into the study satisfied the requirements set forth by the Drug and Cosmetic Act, including being between the ages of 18 and 60, weighing more than 45 kg, and having hemoglobin levels greater than 12.5 gm/dl. Participants who did not provide consent, had aberrant red cell antibodies, tested positive for syphilis, HIV, hepatitis B, hepatitis C, malaria, or were AB blood group donors were excluded from the study.

Sample size calculation

A clinically significant percentage of 46% was utilized, based on previous research, to ascertain the sample size [9-11]. The calculation was based on detecting an expected moderate correlation coefficient (r = 0.30) between titer measurements, with a statistical power (1-β) of 80% and a two-sided alpha level of 0.05. Using these parameters, the minimum required sample size to achieve sufficient statistical power was calculated to be 84 participants. To increase the robustness of our findings and account for multiple subgroup analyses, we ultimately included all 199 donors who met the eligibility criteria and were recruited during the defined study period [12].

ABO isoagglutinin titrations were performed on all group A, B, and O blood donations intended for essential transfusions for hospital patients. Pre-transfusion antibody titration was performed on samples from 199 different donors during the study period, and 1,042 blood donations were made.

Titration method 

The Tube Testing method was used to titrate isoagglutinin A and B in accordance with the Association for the Advancement of Blood and Biotherapies (AABB) technical manual [13]. 0.9% normal-ionic-strength saline (NS), low-ionic-strength solution (LISS), and AB plasma were used as diluents to dilute serum. For all three - normal saline, LISS, and AB plasma - transferring 100 μL from each set of serially diluted serum to a set of 1-12 tubes is necessary for evaluating the IgM isoagglutinin titer at room temperature and the IgG isoagglutinin titer at the indirect agglutination test (IAT) phase. Following the completion of the transfers, each tube's contents are carefully combined to guarantee that the serum is distributed evenly throughout the diluent. The proper testing methods are then carried out in order to assess the serological reactions.

Using the titer as a guide, tubes 1-12 were named 1:1, 1:2, 1:4, 1:8, 1:16, 1:32, 1:64, 1:128, 1:256, 1:512, 1:1024, and 1:2048, respectively. Then we added 50 μL of 3% A1 cells or B cells freshly prepared from random donor units, according to the agglutinin-A or B in plasma, to all tubes. This step is crucial for observing the specific agglutination patterns that indicate the presence of antibodies. Subsequently, all tubes were thoroughly mixed, with one set incubated at room temperature (22-25°C) for 15 minutes, while the second set was incubated at 37°C for 30 minutes. Following the incubation period, each tube in the initial set was centrifuged for one minute at 1000 rpm before examination for agglutinates. The last dilution tube that gave 1+ agglutination macroscopically indicated the titer of the sample. After being reviewed by the technical team and the medical officer, the titers were recorded in the antibody titration report form. 

To reduce inter-operator variability, the titration process was performed by one technician, the results were observed by another technician, and the findings were verified by a medical officer.

After completing the incubation for the second group, the cells in each tube were manually washed three times. Next, we put two drops of commercially manufactured anti-human globulin (AHG) (Tulip Diagnostics, Goa, India) in each tube and centrifuged it for one minute at 1000 rpm. As previously stated, the agglutinates were analyzed, and anti-A or anti-B IgG isoagglutinin titers were reported [14-15].

Statistical analysis

Statistical analysis was conducted with Jamovi 3.2.1 statistics software (www.jamovi.org). Descriptive statistics, including frequencies (n), percentages (%), means (± standard deviation, SD), and medians (interquartile range, IQR), were employed to summarize participant demographics, blood group distributions, and antibody titers. Normality was tested using the Shapiro-Wilk test, which showed that the antibody titer data were not normally distributed. Therefore, we employed the non-parametric Spearman's rank correlation coefficient (r) to assess the strength and significance of the relationship between titer measurements obtained with various diluents. A p-value under 0.05 was deemed statistically significant for all analyses.

## Results

Over a period of 4 months, 226 blood donors took part in the study, and 199 of them fulfilled the inclusion criteria. The exclusion criteria led to the elimination of the remaining 27 from the study. The majority of participants/donors (52.8%) were in the 18-30 years age range, with a mean age of 30.8 years and a standard deviation of 8.16 years. Blood group B (111; 55.77%) comprised the bulk of donors, followed by group A (55; 27.63%) and group O (33; 16.5%). Table 1 shows the study population's age distribution across the blood groups.

**Table 1:** Blood type distribution by age group in the study population

Age (years)	Blood Group A n (%)	Blood Group B n (%)	Blood Group O n (%)	Total n (%)
18–30	27 (49.1%)	59 (53.2%)	19 (57.6%)	105 (52.8%)
31–40	23 (41.8%)	39 (35.1%)	10 (30.3%)	72 (36.2%)
41–50	4 (7.3%)	12 (10.8%)	2 (6.1%)	18 (9.0%)
51–60	1 (1.8%)	1 (0.9%)	2 (6.1%)	4 (2.0%)
Total	55 (100%)	111 (100%)	33 (100%)	199 (100%)

The following sections describe the distribution of median anti-A and anti-B titers in blood types A, B, and O.

Anti-A titer distribution by age in group B

The majority of study participants with titers <64 were in the 18-30 age range (55; 52.9%), and the majority of study participants with titers >64 were in the same age range (4; 57.1%). There was no statistically significant variation in antibody titer or age groups among the various diluents, as indicated by the p-values in Table 2.

**Table 2:** Age-wise distribution of anti-A titer in blood group B with normal saline, LISS and AB plasma Values are expressed as median (interquartile range). Anti-A titers in Group B individuals showed minimal variation with age across all methods, with no statistically significant differences (p > 0.05). LISS: Low ionic strength solution.

Blood Group Antibodies	Age Group (years)	Normal Saline	LISS	AB Plasma
Anti-A in Blood Group B	18–30	16 (8.0–48.0)	32 (12.0–48.0)	16 (8.0–32.0)
	31–40	16 (8.0–16.0)	16 (8.0–32.0)	16 (8.0–48.0)
	41–50	12 (8.0–20.0)	8 (8.0–10.0)	12 (8.0–44.0)
	51–60	16 (16.0–16.0)	16 (16.0–16.0)	16 (16.0–16.0)
χ²		2.91	2.73	2.67
p		0.406	0.435	0.445

Correlation of anti-A titer of blood group B using NS, LISS, and AB plasma

The median anti-A titer in blood group B was 16 (Interquartile Range (IQR) 12-32) in normal saline, 32 (IQR 8-32) in low ionic strength solution (LISS), 16 (IQR 8-32) in AB plasma, and in the AHG phase the titer of anti-A in blood group B is 16 (IQR 8-32), 8 (IQR 8-32), 8 (IQR 4-16) in normal saline, LISS, and AB plasma respectively.

**Figure 1:**
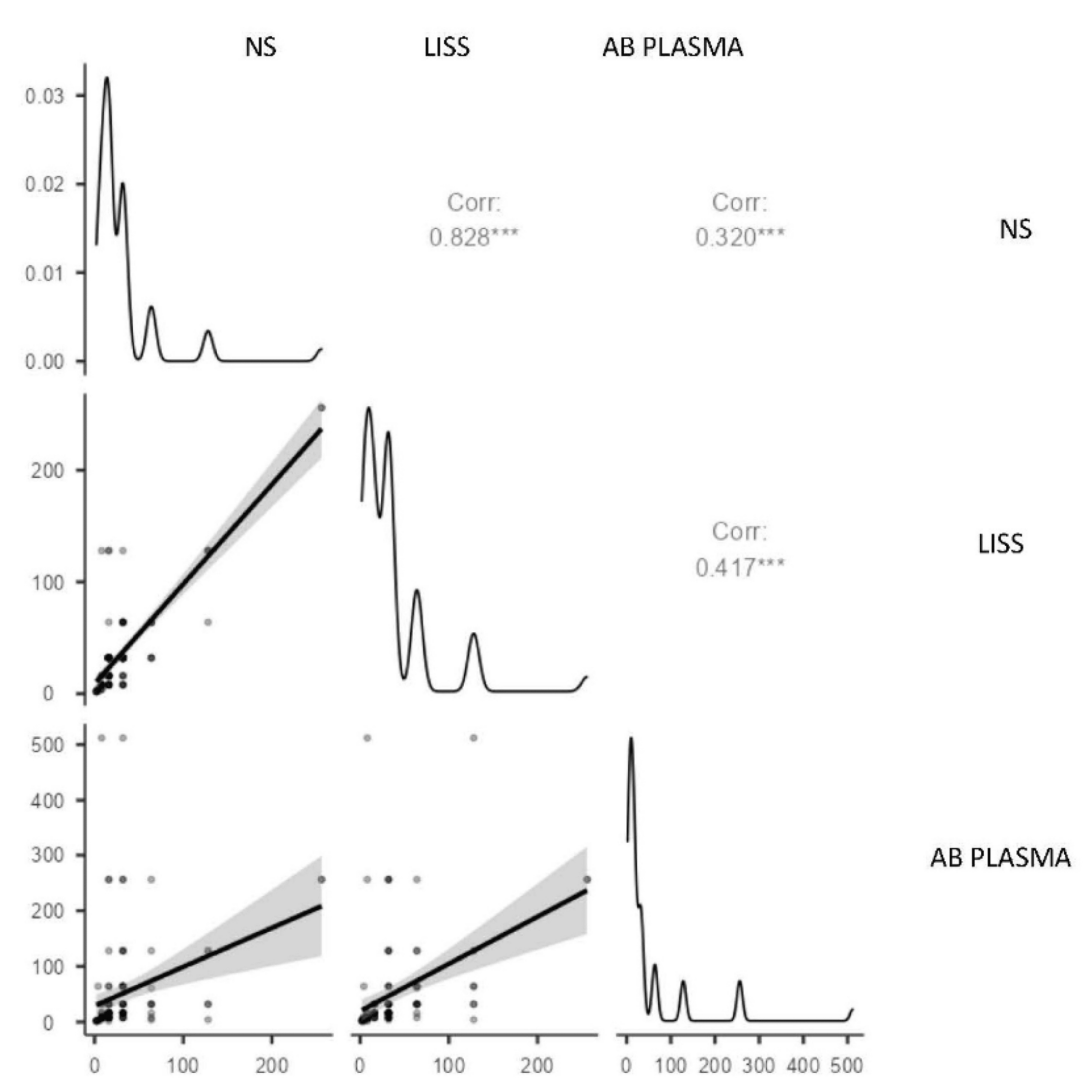
Scatter plot showing correlation of anti-A titer of blood group B (n=111) using NS, LISS & AB plasma NS: normal saline; LISS: low ionic strength solution

The strongest correlation was observed between NS and LISS (r = 0.828, p < .001), indicating high concordance between these two methods. Moderate correlations were found between AB plasma and both NS (r = 0.320) and LISS (r = 0.417), suggesting that AB plasma may yield slightly variable results compared to the other two techniques. All correlations were statistically significant (p < 0.05) as denoted in Figure 1.

Anti-B titer distribution by age in blood group A

The age group of 31-40 years included the majority of the study population with titers <64 (22; 45.8%), while the age group of 18-30 years comprised the majority of the study population with titers >64 (6; 85.7%). The age groups and antibody titers found in the various diluents did not differ statistically significantly, as indicated by the p-values in Table 3.

**Table 3:** Age-wise distribution of anti-B titer in blood group A with normal saline, LISS, and AB plasma Values are expressed as median (interquartile range). Anti-B titers in Group A individuals remained largely consistent across age groups and testing methods. The higher AB plasma titer observed in the 51–60 years group did not reach statistical significance (p > 0.05). LISS: Low ionic strength solution.

BLOOD GROUP Antibodies	Age Group	Normal Saline	LISS	AB PLASMA
Anti-B in Blood Group A	18–30	16 (16.0–32.0)	32 (8.0–32.0)	16 (8.0–64.0)
	31–40	16 (8.0–32.0)	16 (8.0–32.0)	16 (8.0–32.0)
	41–50	16 (16.0–32.0)	32 (16.0–48.0)	16 (8.0–32.0)
	51–60	12 (10.0–14.0)	20 (14.0–26.0)	132 (70.0–194.0)
χ²		2.4776	5.2611	0.0137
p		0.479	0.154	1

Correlation of anti-B titer of blood group A using NS, LISS, and AB plasma

The median anti-B titer in blood group A is 16 (IQR 8-32) in normal saline, 16 (IQR 8-32) in LISS, 16 (IQR 8-32) in AB plasma, and in AHG phase the titer of anti-B in blood group A is 8 (IQR 6-32), 16 (IQR 8-16), 8 (IQR 6-16) in normal saline, LISS, and AB plasma respectively after considering outliers.

**Figure 2:**
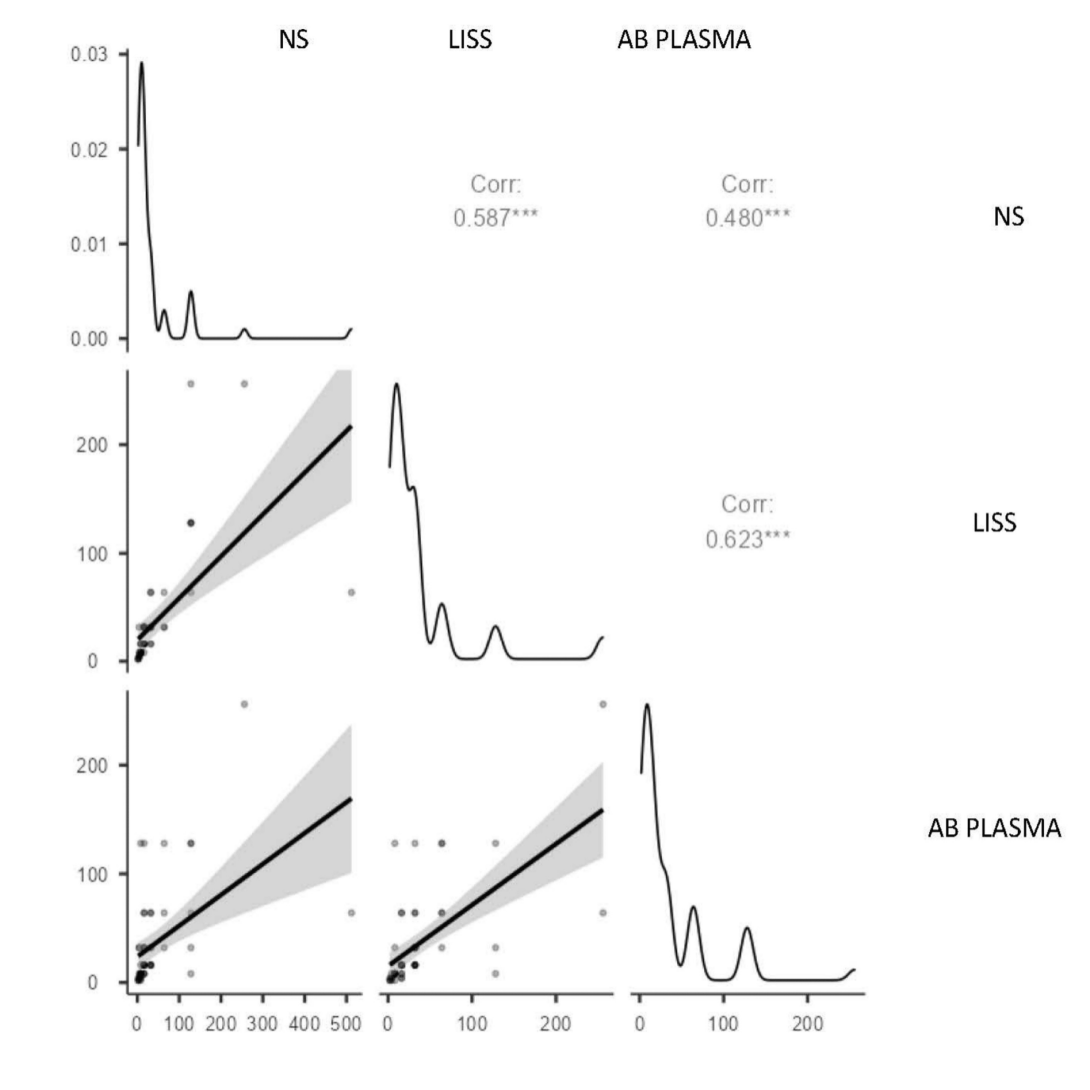
Scatter plot showing correlation of anti-B titer of blood group A using NS, LISS, and AB plasma NS: normal saline; LISS: low ionic strength solution

A moderate positive correlation was observed between NS and LISS (r = 0.587), indicating comparable anti-B titer results using these two media. Correlation between NS and AB plasma was slightly weaker (r = 0.480), while the strongest correlation was between LISS and AB plasma (r = 0.623), as shown in Figure 2.

Anti-A titer distribution by age in blood group O

The majority of study participants with titers <64 were in the 18-30 age range (11; 55.0%), whereas the majority of study participants with titers >64 were in the same age range (8; 61.5%). Using various diluents, there was no statistically significant difference in antibody titers or age groups; the p-values are displayed in Table 4.

**Table 4:** Age-wise distribution of anti-A titer in group O with normal saline, LISS, and AB plasma Values are expressed as median (interquartile range). Anti-A titers in Group O individuals were higher compared to Group B, particularly in younger age groups; however, no statistically significant variation was observed across age groups (p > 0.05). LISS: Low ionic strength solution.

Blood Group Antibodies	Age Group	Normal Saline	LISS	AB Plasma
Anti-A In Blood Group O	18–30	64 (16.0–128.0)	64 (32.0–128.0)	32 (16.0–64.0)
	31–40	96 (64.0–224.0)	128 (128–128)	64 (64.0–112.0)
	41–50	24 (20.0–28.0)	24 (20.0–28.0)	96 (80.0–112.0)
	51–60	24 (20.0–28.0)	80 (56.0–104.0)	80 (56.0–104.0)
χ²		3.44	3.46	2.8
p		0.328	0.326	0.423

Correlation of anti-A titer of blood group O using NS, LISS, and AB plasma

The median anti-A titer in blood groups O is 64 (IQR 16-128) in normal saline, 128 (IQR 32-128) in LISS, 64 (IQR 16-128) in AB plasma, and in the AHG phase the titer of anti A in blood group B is 32 (IQR 8-64), 64 (IQR 16-64), 32 (IQR 16-64) in normal saline, LISS, and AB plasma respectively.

**Figure 3:**
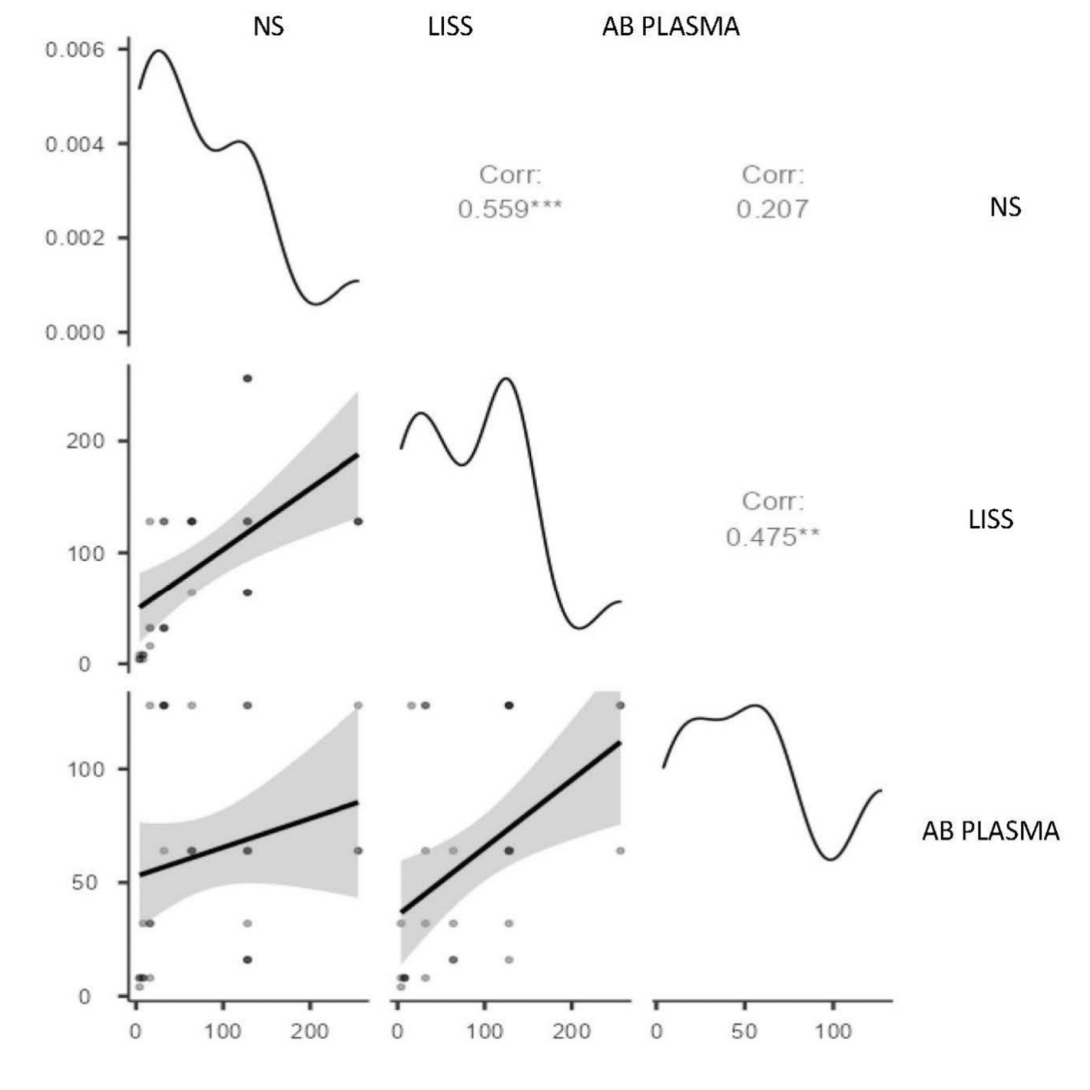
Scatter plot showing correlation of anti-A titer of blood group 0 using NS, LISS , and AB plasma NA: normal saline; LISS: low ionic strength solution

NS vs LISS showed a moderate and significant correlation (r = 0.559), indicating good agreement between these two methods. In contrast, as demonstrated in Figure 3, the correlation between NS and AB Plasma was weak and non-significant (r = 0.207, p = 0.247), whereas the connection between LISS and AB Plasma was substantial and significant (r = 0.475, p = 0.005), indicating superior consistency.

Anti-B titer distribution by age in blood group O

The study found that the majority of participants with titers below 64 were in the 18-30 age group (16 out of 30; 59.3%), and that the majority of participants with titers above 64 were in the same age group (three out of 56.0%). The results show that there was no significant difference in antibody titers or age groups when using different diluents. The p-values are presented in Table 5.

**Table 5:** Age-wise distribution of anti-B titer in blood group O with normal saline, LISS, and AB plasma Values are expressed as median (interquartile range). Anti-B titers in Group O individuals demonstrated minor fluctuations across age groups and methods, but these were not statistically significant (p > 0.05). LISS: Low ionic strength solution.

Blood Group Antibodies	Age Group	Normal Saline	LISS	AB PLASMA
Anti-B in Blood Group O	18–30	32 (16.0–64.0)	64 (24.0–64.0)	32 (16.0–128.0)
	31–40	64 (32.5–112.0)	64 (40.0–64.0)	64 (40.0–64.0)
	41–50	12 (10.0–14.0)	10 (7.0–13.0)	80 (56.0–104.0)
	51–60	20 (14.0–26.0)	36 (22.0–50.0)	48 (40.0–56.0)
χ²		5.119	4.693	0.283
p		0.163	0.196	0.963

Correlation of anti-B titer of blood group O using NS, LISS, and AB plasma

The median anti-B titer in blood group O is 32 (IQR 16-64) in normal saline, 64 (IQR 16-64) in LISS, 64 (IQR 32-128) in AB plasma, and in the AHG phase the titer of anti-B in blood group O is 32 (IQR 32-64), 32 (IQR 16-32), 32 (IQR 8-64) in normal saline, LISS, and AB plasma, respectively.

**Figure 4:**
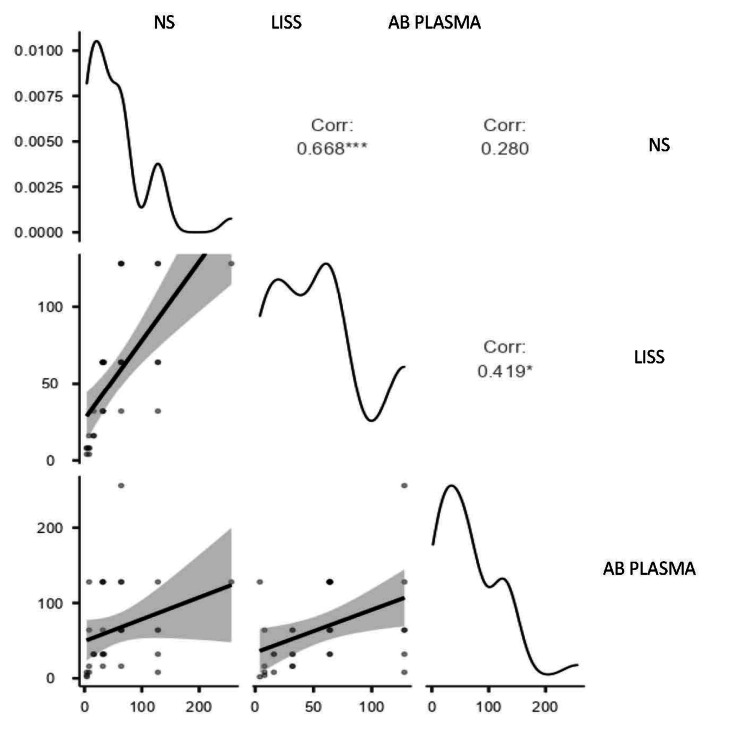
Scatter plot showing correlation of anti-B titer of blood group O using NS, LISS , AB plasma NS: normal saline; LISS: low ionic strength solution

NS vs LISS exhibited a robust and statistically significant correlation (r = 0.668, p < .001), demonstrating good agreement. As shown in Figure 4, there was a moderately significant correlation (r = 0.419, p = 0.015) between LISS and AB plasma and a weak and non-significant correlation (r = 0.280, p = 0.115) between NS and AB plasma. This suggests that LISS and AB plasma are more consistent.

## Discussion

Estimating the isoagglutinin A and B titers in the donors from blood groups A, B, and O using various diluents was the primary objective of the current investigation.

The anti-B titer showed the strongest correlation between NS and LISS in people with blood group A (r = 0.828, p <.001), suggesting that these two techniques are highly compatible. Both NS (r = 0.320) and LISS (r = 0.417) showed weaker correlations with AB plasma, while NS and LISS (r = 0.587) showed a moderately positive association with anti-A titer in blood group B individuals, suggesting similar anti-A titer outcomes utilizing these two media. There was a somewhat weaker correlation (r = 0.480) between NS and AB plasma.

Distribution of IgM titer of A and B agglutinins

Most investigations have regarded antibody titers of >64 as high titers [9-10]. Based on the previously mentioned investigations, the current study employed a cut-off titer of 64 for the analysis. Among the blood group A patients, 48 (87.27%) exhibited IgM anti-B titers of ≤64, whereas seven (12.72%) displayed titers of >64. 104 individuals (93.69%) with blood Group B exhibited an anti-A titer of ≤64, while seven individuals (6.30%) had a titer >64.

While IgM agglutinin B titers of >64 were six (18.18%), IgM agglutinin A titers of >64 were 13 (39.39%) in blood group O individuals. Consequently, a greater proportion of donors exhibited IgM agglutinin A and B titers of less than 64.

Research by Hashim M et al. found that IgM agglutinin B titers of less than 64 were found in 73.4% of blood group O persons and more than 64 in 26.6% of them, which is comparable to the current study [9]. In line with the current investigation, 52.85% of cases had titers of less than 64, while 47.15% had titers of more than 64, according to Kavallierou L et al. [10]. Further research by Gopal S et al. similarly supported the current study's findings, which showed that titers of >64 were observed in 38% of cases [15].

Gopal S et al. found that in blood Group O, isoagglutinin A titers of less than 64 were 59%, whereas titers of more than 64 were 41%. These findings were comparable to the current investigation [15].

The antibody titers in all blood groups were evaluated in this first study from Central India, even though other investigations by Tendulkar AA et al. have focused on blood Group O. Using alternative diluents did not result in statistically significant differences in antibody titers or age groups. This was found to be similar to the research conducted by Tendulkar AA et al., which indicated that titer levels declined as age increased, suggesting that levels and age had an inverse connection [16].

Kannan S et al. found that in blood group O, isoagglutinin A titers of less than 64 were 60.57%, whereas titers of more than 64 were 39.43%. These findings were comparable to the current investigation [17]. The titer age distribution in this investigation was similar to that in the study by Kumar K et al. [18].

IgM agglutinin B titers in blood group A by age

Of blood group A members, 22 (45.8%) had titers of 64 in the 18-30 years age range. This aligned with the research done by Kumar K. et al. [18].

IgM agglutinin A titers in blood group B by age

Of those in blood group B, 55 (52.90%) had IgM anti-A titers of less than 64 in the 18-30 years age range, while 4 (57.10%) had titers of more than 64 in the same age range.

Blood group O titer distribution by age

Of those in blood Group O, 11 (55.0%) had IgM anti-A titers of less than 64 in the 18-30 years age range, while eight (61.5%) had titers of greater than 64 in the same age range. Current research findings align with those of a study by Kumar K. et al. [18]. Blood group O study participants with IgM agglutinin B titers of less than 64 in the 18-30 years age range comprised 16 (59.3%), while three (50.0%) had titers of more than 64 in the same age group. The findings of the current study were similar to those of the South Rajasthan study [18].

The findings of the current investigation were in line with Thattanon P et al., who didn’t find any correlation between antibody titers and age [19].

According to de França ND et al., titers of less than 128 were observed in 90.71% of cases, whereas titers of greater than 128 were found in 9.29% of cases. They reported a greater proportion of people with low titers [20].

In contrast to the Datta SS et al. study, which found that titers were greater in the 40-49 years age group [21], this study's blood group A participants had median agglutinin B titers that were comparable until they were 50 years old, at which point they started to decrease.

Agglutinin A and agglutinin B titer correlation with different diluents

Blood group O, NS, and LISS agglutinin A titres showed a moderate and statistically significant correlation (r = 0.559, p < 0.001), indicating good agreement between the two methods. In contrast, NS vs. AB Plasma showed a poor and non-significant correlation (r = 0.207, p = 0.247), whereas LISS vs. AB Plasma showed a moderate, significant correlation (r = 0.475, p = 0.005), suggesting better consistency. For agglutinin B titre of blood group O, the results showed a strong and statistically significant correlation (r = 0.668, p = 0.115) between NS and LISS, signifying a remarkable concordance. The association between LISS and AB Plasma was moderate and significant (r = 0.419, p = 0.015), demonstrating greater consistency than the weak and non-significant correlation between NS and AB Plasma (r = 0.280, p = 0.115).

## Conclusions

Individuals with blood type A exhibited the most significant correlation between NS and LISS, indicating a pronounced sensitivity to LISS as a diluent. The anti-A titer values were comparable while utilizing these two media. NS and LISS had a moderately positive correlation with anti-A titer in persons with blood group B. There was a strong correlation between the two approaches regarding blood group O, NS, and LISS agglutinin A titers, as well as for agglutinin B titer in blood group O. All blood centers should evaluate antibody titers for each donor across multiple blood groups to maintain a database for both group-specific and non-group-specific transfusions. Recipients belonging to the same blood group should not receive transfusions from non-group-specific donors whose titer value exceeds 64. Acute hemolytic transfusion reactions have been seen in patients receiving non-group-specific platelet concentrate with antibody titers of 64 or higher, although the clinical outcome is greatly influenced by the volume of blood transfused. LISS serves as a more sensitive diluent for antibody titration in high-risk scenarios, such as hematopoietic stem cell transplants, high-risk pregnancies, or prior transfusion reactions, to mitigate the likelihood of future events.
